# Supplementary figures and images for: Endozoochory by the cooperation between beetles and ants in the holoparasitic plant Cynomorium songaricum in the deserts of Northwest China
Source: PLoS One. 2025 Mar 11;20(3):e0319087. doi: 10.1371/journal.pone.0319087 (PMC11896033; doi:10.1371/journal.pone.0319087)

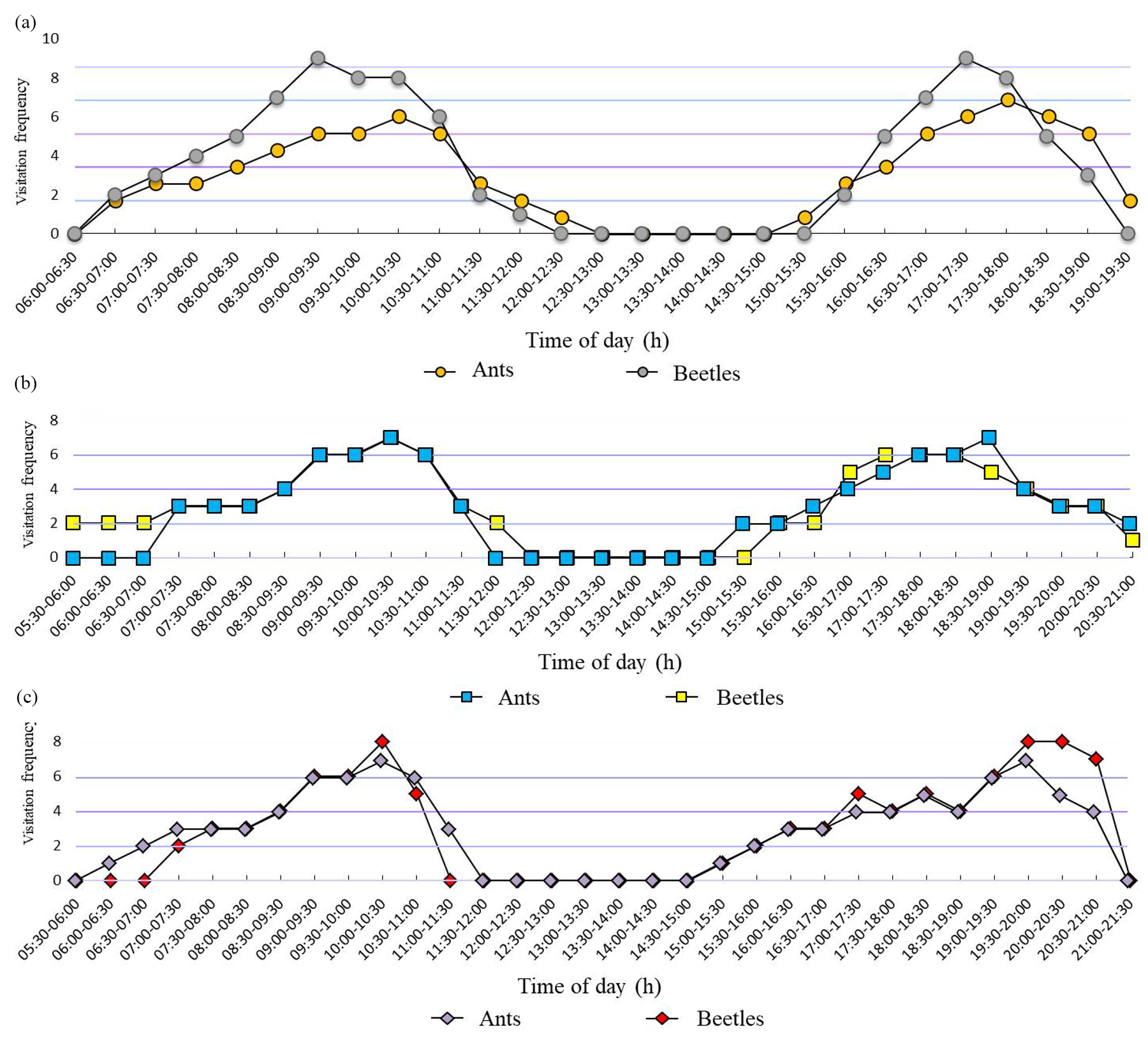

Supplement: S1 Fig — (a) Jilantai Town; (b) Yingen Sumu; (c) Ejina Banner. The study of three research sites found that beetles mainly nibbled on Suoyang in the morning (06:30-11:30) and afternoon (15:30-19:00). During the day (12:00-15:30), activity decreases sharply and the beetles disappear into the sand. Ants mainly consume seeds in the morning (06:30-12:30) and afternoon (15:00-19:30). During the day (12:30-15:00), activity decreases sharply, diving into the nest (Fig 3 and Fig 4). The difference of visiting time between ants and beetles in different research sites is related to the temperature of the research sites. (TIF) [file pone.0319087.s001.tif]

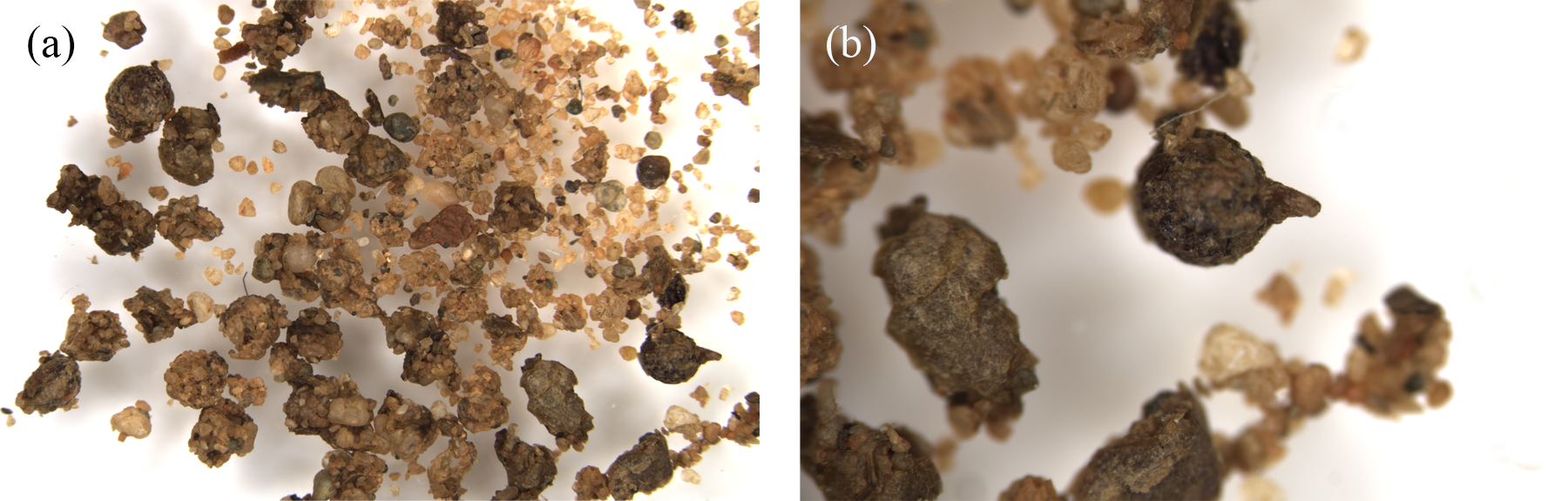

Supplement: S2 Fig — (a) M. semenowi feces; (b) C. songaricum seeds; The area circled in red is C. songaricum seeds. (a), Bar = 2 mm; (b), Bar = 1 mm. (TIF) [file pone.0319087.s002.tif]

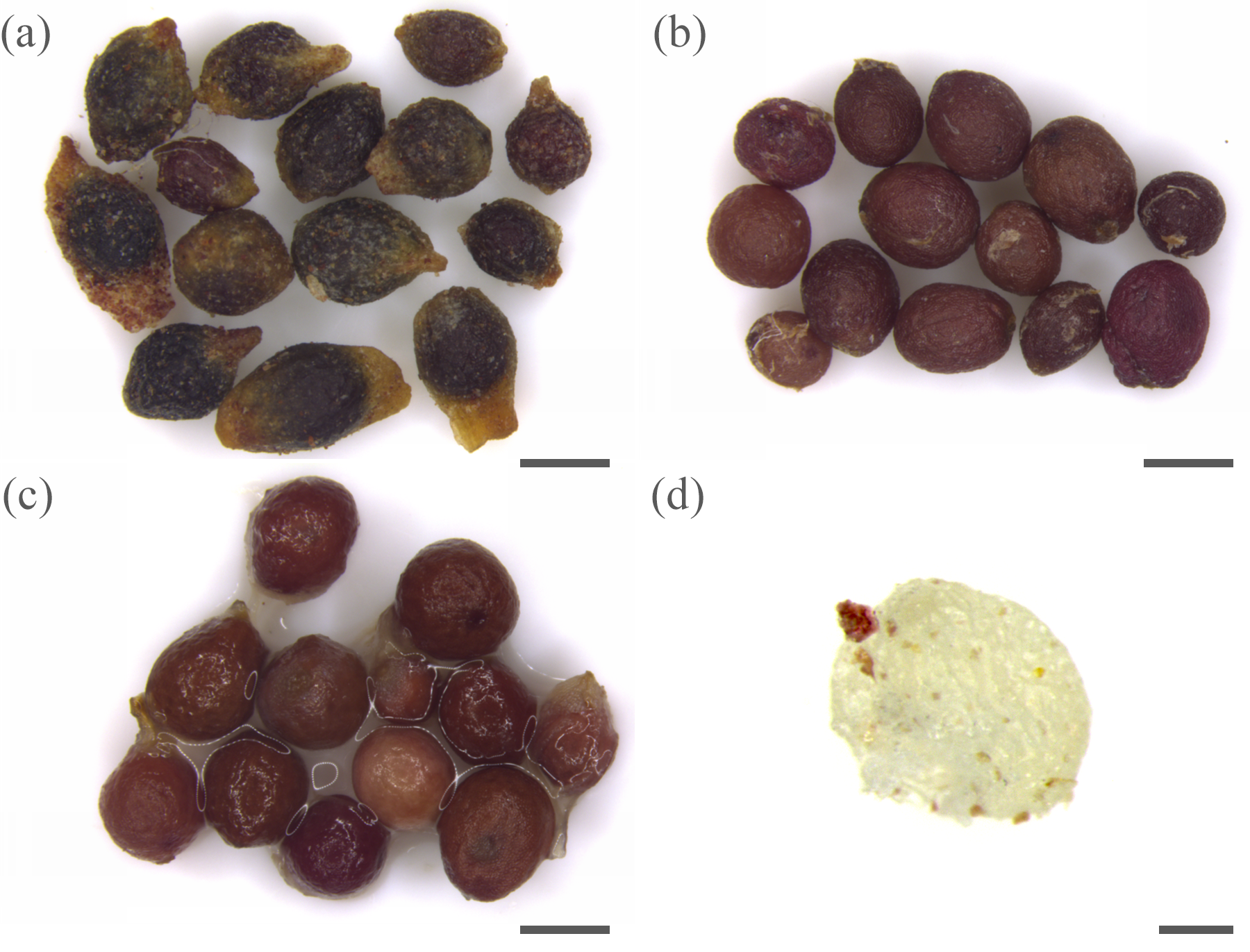

Supplement: S3 Fig — (a) Natural seeds; (b) non-elaiosome seeds; (c) elaiosome comes into contact with water and wraps C. songaricum seeds; (d) Embryo. (a), (b), (c), Bars = 1 mm. (d), Bar = 500 μm. The elaiosome of C. songaricum seeds. When fruits are brown and dried, the dry pulp seems to play the role of an elaiosome. (TIF) [file pone.0319087.s003.tif]

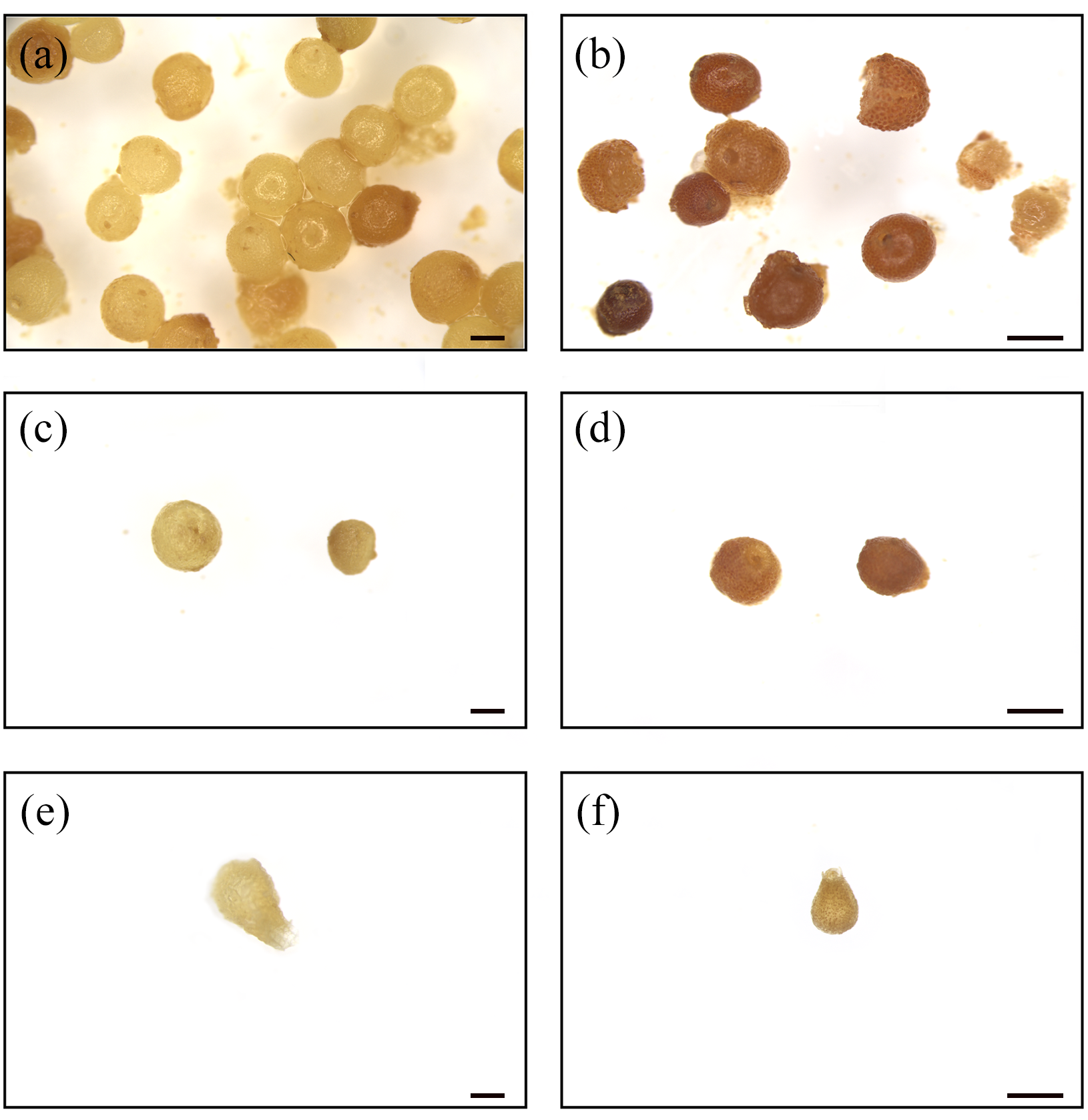

Supplement: S4 Fig — (a-b) Seed embryos in feces after 12 hours; (c-d) Seed embryos in feces after 24 hours; (e-f) Seed embryos in feces after 48 hours; (a, c, e) Unstained seed embryos; (b, d, f) Stained seed embryos. (a), (c), (e), Bars = 2 mm. (b), (d), (f), Bars = 1 mm. (TIF) [file pone.0319087.s004.tif]

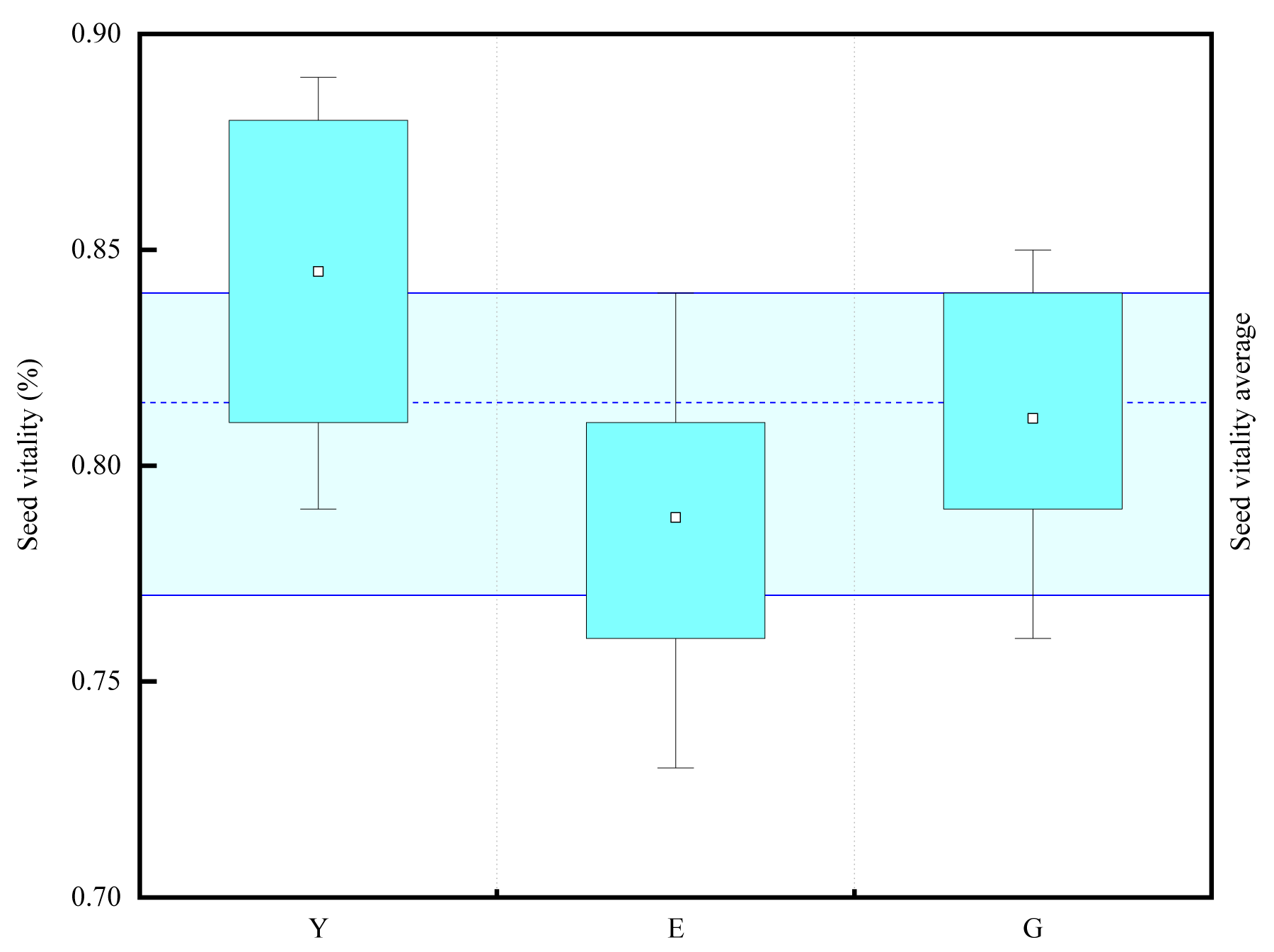

Supplement: S5 Fig — (Y) Yingen Sumu; (E) Ejina Banner; (J) Jilantai Town. The results of the seed vigor test at the three research sites showed that under natural conditions, the vigor of C. songaricum seeds at the three research sites was above 75%, including Yingen Sumu (84.5 ± 3.60%), Jilantai Town (81.1 ± 3.07%), and Ejin Banner (78.8 ± 3.46%). (TIF) [file pone.0319087.s005.tif]
