# Supplementary material for: Endozoochory by the cooperation between beetles and ants in the holoparasitic plant Cynomorium songaricum in the deserts of Northwest China
Source: PLoS One. 2025 Mar 11;20(3):e0319087. doi: 10.1371/journal.pone.0319087 (PMC11896033; doi:10.1371/journal.pone.0319087)
Supplement: S1 Table — (DOCX) [file pone.0319087.s006.docx]

S1 Table: **Study sites**

| **Study site** | **Observation points** | **Longitude** | **Latitude** | **Altitude** |
| --- | --- | --- | --- | --- |
| Ejina | E1 | 100.66505 | 41.41956 | 1001.30 |
|  | E2 | 100.66660 | 41.41244 | 1000.60 |
|  | E3 | 100.667468 | 41.416322 | 997.60 |
|  | E4 | 100.668005 | 41.416883 | 997.60 |
|  | E5 | 100.668325 | 41.419009 | 997.60 |
| Yingen | Y1 | 104.71048 | 41.383637 | 758.70 |
|  | Y2 | 104.7102 | 41.3855 | 752.30 |
|  | Y3 | 104.710418 | 41.383705 | 749.30 |
|  | Y4 | 104.710418 | 41.383705 | 749.30 |
|  | Y5 | 104.708718 | 41.385677 | 756.90 |
| Jilantai | J1 | 105.42191 | 39.20458 | 1162.00 |
|  | J2 | 105.41425 | 39.23112 | 1138.20 |
|  | J3 | 105.42145 | 39.20421 | 1163.20 |
|  | J4 | 105.42345 | 39.21845 | 1152.26 |
|  | J5 | 105.42256 | 39.20158 | 1163.25 |
