# Supplementary material for: Endozoochory by the cooperation between beetles and ants in the holoparasitic plant Cynomorium songaricum in the deserts of Northwest China
Source: PLoS One. 2025 Mar 11;20(3):e0319087. doi: 10.1371/journal.pone.0319087 (PMC11896033; doi:10.1371/journal.pone.0319087)
Supplement: S2 Table — (DOCX) [file pone.0319087.s007.docx]

**S2 Table. The time, place and number of samples observed by visitors to the natural population of *C. songaricum.***

| Study site | Date | | | Time  (h) |
| --- | --- | --- | --- | --- |
|  | Year | Month | Day |  |
| Ejina | 2021 | 6 | 8,9 | 84 |
|  |  | 7 | 15-17 |  |
|  |  | 8 | `17,18 |  |
|  | 2022 | 6 | 7-9 | 108 |
|  |  | 7 | 11-13 |  |
|  |  | 8 | 10-12 |  |
| Jilantai | 2021 | 6 | 15,16 | 88 |
|  |  | 7 | 11-13 |  |
|  |  | 8 | 13-15 |  |
|  | 2022 | 6 | 16-18 | 132 |
|  |  | 7 | 5-7 |  |
|  |  | 8 | 5-8 |  |
| Yingen | 2021 | 6 | 11,12 | 112 |
|  |  | 7 | 19-21 |  |
|  |  | 8 | 20-22 |  |
|  | 2022 | 6 | 11-13 | 112 |
|  |  | 7 | 17,18 |  |
|  |  | 8 | 16-18 |  |
| Total |  |  | 50 | 636 |
