# Supplementary material for: Endozoochory by the cooperation between beetles and ants in the holoparasitic plant Cynomorium songaricum in the deserts of Northwest China
Source: PLoS One. 2025 Mar 11;20(3):e0319087. doi: 10.1371/journal.pone.0319087 (PMC11896033; doi:10.1371/journal.pone.0319087)
Supplement: S3 Table — (DOCX) [file pone.0319087.s008.docx]

**S3 Table. The number of seeds that an *M. desertora* transports from the fleshy stem of *C. songaricum* back to the nest within a day.**

| **Repeat** | **Time/min** |
| --- | --- |
| 1 | 28.5 |
| 2 | 24.7 |
| 3 | 12.2 |
| 4 | 9.3 |
| 5 | 16.1 |
| 6 | 13.8 |
| 7 | 10.2 |
| 8 | 11.4 |
| 9 | 15.7 |
| 10 | 15.3 |
| 11 | 9.9 |
| 12 | 10.6 |
| 13 | 12.8 |
| 14 | 12.9 |
| 15 | 13.3 |
| 16 | 12.9 |
| 17 | 6.1 |
| 18 | 15.2 |
| 19 | 11.8 |
| 20 | 16.5 |
| 21 | 10.1 |
| 22 | 12.5 |
| 23 | 28.5 |
| 24 | 10.2 |
| 25 | 14.2 |
| 26 | 6.9 |
| 27 | 11.7 |
| 28 | 15.4 |
| 29 | 10.1 |
| 30 | 28.6 |
| AVG | 14.25 |
| SD | 5.91 |
