# Supplementary material for: Endozoochory by the cooperation between beetles and ants in the holoparasitic plant Cynomorium songaricum in the deserts of Northwest China
Source: PLoS One. 2025 Mar 11;20(3):e0319087. doi: 10.1371/journal.pone.0319087 (PMC11896033; doi:10.1371/journal.pone.0319087)
Supplement: S4 Table — (DOCX) [file pone.0319087.s009.docx]

**S4 Table.** **The average time it takes for an *M. desertora* to bite off a seed from the fleshy stem of *C. songaricum* and carry it back to the nest.**

| **Repeat** | **number/day** |
| --- | --- |
| 1 | 172 |
| 2 | 175 |
| 3 | 120 |
| 4 | 108 |
| 5 | 115 |
| 6 | 105 |
| 7 | 148 |
| 8 | 125 |
| 9 | 127 |
| 10 | 130 |
| 11 | 135 |
| 12 | 126 |
| 13 | 121 |
| 14 | 145 |
| 15 | 165 |
| 16 | 151 |
| 17 | 138 |
| 18 | 146 |
| 19 | 156 |
| 20 | 165 |
| 21 | 130 |
| 22 | 119 |
| 23 | 120 |
| 24 | 125 |
| 25 | 145 |
| 26 | 146 |
| 27 | 161 |
| 28 | 149 |
| 29 | 241 |
| 30 | 219 |
| AVG | 144.27 |
| SD | 29.94 |
