# Supplementary material for: Endozoochory by the cooperation between beetles and ants in the holoparasitic plant Cynomorium songaricum in the deserts of Northwest China
Source: PLoS One. 2025 Mar 11;20(3):e0319087. doi: 10.1371/journal.pone.0319087 (PMC11896033; doi:10.1371/journal.pone.0319087)
Supplement: S5 Table — (DOCX) [file pone.0319087.s010.docx]

**S5 Table. The average time it takes for a *M. semenowi* to bite off seeds from the fleshy stem of *C. songaricum.***

| Repeat | Time/s |
| --- | --- |
| 1 | 10.5 |
| 2 | 14.5 |
| 3 | 20 |
| 4 | 19 |
| 5 | 15 |
| 6 | 14 |
| 7 | 10 |
| 8 | 15 |
| 9 | 14 |
| 10 | 16 |
| 11 | 14 |
| 12 | 15 |
| 13 | 10 |
| 14 | 12 |
| 15 | 12 |
| 16 | 11.2 |
| 17 | 15.5 |
| 18 | 20 |
| 19 | 16.5 |
| 20 | 15 |
| 21 | 12 |
| 22 | 11 |
| 23 | 12.5 |
| 24 | 15.5 |
| 25 | 16 |
| 26 | 13 |
| 27 | 11 |
| 28 | 11.5 |
| 29 | 12 |
| 30 | 10.5 |
| AVG | 13.81 |
| SD | 2.80 |
