# Supplementary material for: Endozoochory by the cooperation between beetles and ants in the holoparasitic plant Cynomorium songaricum in the deserts of Northwest China
Source: PLoS One. 2025 Mar 11;20(3):e0319087. doi: 10.1371/journal.pone.0319087 (PMC11896033; doi:10.1371/journal.pone.0319087)
Supplement: S6 Table — (DOCX) [file pone.0319087.s011.docx]

**S6 Table. Scanning electron microscope observation of seeds in *M. semenowi* feces.**

| **The number of rounds in the feeding experiment** | **The number of damaged seeds** |
| --- | --- |
| 1st rnd | 3 |
| 2st rnd | 2 |
| 3st rnd | 2 |
| 4st rnd | 2 |
| 5st rnd | 1 |
| 6st rnd | 1 |
| AVG | 1.83 |
| SD | 0.8 |
