# Supplementary material for: Endozoochory by the cooperation between beetles and ants in the holoparasitic plant Cynomorium songaricum in the deserts of Northwest China
Source: PLoS One. 2025 Mar 11;20(3):e0319087. doi: 10.1371/journal.pone.0319087 (PMC11896033; doi:10.1371/journal.pone.0319087)
Supplement: S7 Table — (DOCX) [file pone.0319087.s012.docx]

**S7 Table.** **Anatomical observation of seeds in the *M. semenowi* digestive tract.**

| **Feeding Methods** | **6 h** | **12 h** | **24 h** | **48 h** |
| --- | --- | --- | --- | --- |
| Individually raised | 25 | 3 | 1 | 0 |
|  | 22 | 4 | 2 | 0 |
|  | 24 | 3 | 2 | 0 |
| AVG | 23.67 | 3.33 | 1.67 | 0.00 |
| SD | 1.53 | 0.58 | 0.58 | 0.00 |
| Mixed Feeding | 28 | 8 | 5 | 2 |
|  | 31 | 6 | 4 | 1 |
|  | 35 | 5 | 3 | 0 |
| AVG | 31.33 | 6.33 | 4.00 | 1.00 |
| SD | 3.51 | 1.53 | 1.00 | 1.00 |
