# Supplementary material for: Endozoochory by the cooperation between beetles and ants in the holoparasitic plant Cynomorium songaricum in the deserts of Northwest China
Source: PLoS One. 2025 Mar 11;20(3):e0319087. doi: 10.1371/journal.pone.0319087 (PMC11896033; doi:10.1371/journal.pone.0319087)
Supplement: S8 Table — (DOCX) [file pone.0319087.s013.docx]

**S8 Table. Visiting frequency of *M. semenowi* and *M. desertora* to the *C. songaricum* infructescence under different handles.**

| Time | Open infructesence | Odor | Odor control | Sight | Sight control |
| --- | --- | --- | --- | --- | --- |
| 05:30-07:30 | 36.00 | 25.00 | 7.00 | 21.00 | 14.00 |
| 07:30-09:30 | 46.00 | 40.00 | 24.67 | 28.00 | 15.67 |
| 09:30-12:00 | 45.00 | 42.00 | 23.67 | 29.00 | 15.67 |
| 12:00-14:30 | 0.00 | 0.00 | 0.00 | 0.00 | 0.00 |
| 14:30-16:30 | 40.00 | 32.00 | 17.67 | 21.00 | 16.33 |
| 16:30-18:30 | 49.00 | 36.00 | 29.00 | 25.00 | 18.33 |
| 18:30-20:00 | 26.00 | 20.00 | 7.00 | 9.00 | 4.00 |
| 20:00-22:00 | 15.00 | 9.00 | 3.00 | 3.00 | 3.00 |
| AVG | 32.13 | 25.50 | 14.00 | 17.00 | 10.88 |
| SD | 17.25 | 15.06 | 11.09 | 11.40 | 7.26 |
| SUM | 257.00 | 204.00 | 112.00 | 136.00 | 87.00 |
