# Supplementary material for: Endozoochory by the cooperation between beetles and ants in the holoparasitic plant Cynomorium songaricum in the deserts of Northwest China
Source: PLoS One. 2025 Mar 11;20(3):e0319087. doi: 10.1371/journal.pone.0319087 (PMC11896033; doi:10.1371/journal.pone.0319087)
Supplement: Attachment — Submitted filename: PONE-D-24-28571_reviewer.docx [file pone.0319087.s014.docx]

**PLOS ONE**

**Manuscript Number**: PONE-D-24-28571

**Article Type:** Research Article

**Full Title**: Endozoochory by the cooperation between beetles and ants in the holoparasitic plant *Cynomorium songaricum* in the deserts of Northwest China

**General comments to the authors:**

The manuscript needs, in my opinion, minor corrections.

Here are some critical points that need to be addressed in the revised version of the manuscript:

**Introduction**: Authors need to insert more recent information (articles published) on the subjects covered.

**Material and Methods**: Author should clear all the necessary queries and changes recommended (especially experiments design and statistical analysis) because experiment design is too important in research.

**Results:** Some paragraphs in the results section need to be rewritten or clarified for better comprehension.

**Conclusion:** add conclusion section with the mains finding.

**All specifics comment are grouped in the table below**

| Line | Comments / questions |
| --- | --- |
| 38 | Please delete Kingdom |
| 42 | Parasitical plants are divided into hemiparasite and holoparasite plants according to their light and ability. Please add more details |
| 72-73 | Please add citation to this sentence |
| 80 | Please put proper word instead management |
| 87 | Delete “Our” |
| 114 | - Please add the name of three sites were research was conducted with a brief description of each one. - Please add a map showing the three sites. |
| 115 | What do you mean by Sutra? |
| 117 to 120 | “The C. songaricum population is a wild population that relies on Nitrariaceae as its host plant. The C. songaricum seeds were in contact with or closely located in the ground, suggesting that they could be consumed by animals that live near to the ground” please remove this sentences from Materials and Methods to Introduction. |
| 121 | Please update recent citation.  Why did you choose remote camera for investigation?  I think investigation took a long time? Can you explain why |
| 128 | Please add measures under bars for photos. |
| 131 | - Throughout, use the species name of studied insects instead of beetles and ants. |
| 133 | - To avoid repetition put “interactions between C. songaricum and the two studied insects”. Instead of “, interactions between C. songaricum and beetles, and C. songaricum and ants” |
| 135 to 141 | Pease add more details concerning beetles foraging  Experiment design, indicate the following information:   - The number of visits you did in 2021 and 2022 - The frequency of visits (weekly, Monthly...etc) - Did you visit the three sites during one day or each day you visit one site. - Duration of each visit to catch coleoptera. Did you use a standard duration? - Were beetles captured equally in the three sites? indicate the differences if any |
| 146-147 | Please mention the number of repetition to measure this duration.  How did you calculate the number of seeds carried out the anthill? |
| 135  To  147 | Please remove all results from this section. |
| 152-153 | Please rephrase the sentence: 30 beetles captured in the wild were placed in the same breeding room in groups of 10, for a total of three breeding rooms (n=30). |
| 148  To  165 | Please mention breeding parameters in laboratory for both ants and coleoptera. |
| 181  To 192 | Please mention the number of repetitions adopted in experiment of odor and view signals. |
| 192 | Before results section put a paragraph of statistic’s analysis. Mention the tests used. |
| 197 | Throughout, use the species name of studied insects instead of beetles and ants. |
| 199 | To avoid repetition please rephrase the sentence “the seeds are often visited by ants, and ants usually carry the seeds to their nests” |
| 204 | Please rephrase the title of table1.  Delete the name of studied sites in the title |
| 219 | Fruit or seeds? If fruit, how many seeds in average contain? |
| 222 | What do you mean by “most recently 20 cm”? |
| 225 | How did you calculate 47.4%. You didn’t mention the protocol in Material & methods section? |
| 228 | There is a contradiction between the number of seeds found in excrement and the maximum number of seeds eaten by beetles (line 217) |
| 229 to 238 | - You analyzed the obtained data in global manner (you compare only rate of excreted seeds) - Please rewrite this paragraph to mention statistical test used and how you performed analysis. - You should compare the number of seeds excreted between the two feeding systems adopted (individual and mixed breeding) while considering beetles as repetition. |
| 239 | Please make the table title short as possible. |
| 243 | - Table 2: you mentioned in experiment that beetles reared individually were feed only 10 seeds. So, I think ungnawed seeds and gnawed seeds will be less than 10. - Please how did you calculate the number of seeds in beetle feces under different feeding system? |
| 252 | Please replace “the beetles 24 h after feeding, the seeds are feces” by the beetles 24 h after feeding, the seeds are found in feces |
| 257 | Please delete (and exposed) |
| 258-265 | Please move this paragraph to discussion section. |
| 271 | Please mention the dissection experiment in Methods section |
| 310-314 | Please rewrite this paragraph to avoid repetition of some words. |
| 335 to  351 | Please mention how did you calculate the number of visit? And for how long?  I recommend the authors to calculate number or the frequency of visits for each insect, and then compare the number of visits between the two visitors.  Please mention how did you performed statistical analysis and which test did you used |
