# Supplementary material for: Endozoochory by the cooperation between beetles and ants in the holoparasitic plant Cynomorium songaricum in the deserts of Northwest China
Source: PLoS One. 2025 Mar 11;20(3):e0319087. doi: 10.1371/journal.pone.0319087 (PMC11896033; doi:10.1371/journal.pone.0319087)
Supplement: Attachment — Submitted filename: Response to Reviewers.doc [file pone.0319087.s015.doc]

**Response to reviewers**

Dear editor and reviewers,

Thank you for offering us an opportunity to improve the quality of our submitted manuscript entitled “Endozoochory by the cooperation between beetles and ants in the holoparasitic plant *Cynomorium songaricum* in the deserts of Northwest China” (PONE-D-24-28571). We sincerely thank the reviewer for the valuable comments and constructive suggestions, which have significantly contributed to improving the quality of our manuscript. We appreciate the reviewer’s time and effort in carefully evaluating our work.

In response to the comments provided, we have carefully revised the manuscript. Below is a summary of the changes made to address the main concerns:

**Responses to Reviewer #1：**

**Introduction:** Authors need to insert more recent information (articles published) on the subjects covered.

**Response:** Thank you for your valuable comments. In response to the reviewer's suggestions, we have updated the introduction section by incorporating recent literature to ensure the discussion reflects the latest advancements in the relevant research areas.

**Materials and Methods:** Author should clear all the necessary queries and changes recommended (especially experiments design and statistical analysis) because experiment design is too important in research.

**Response:** We gratefully appreciate your valuable comment. We have clarified the experimental design and statistical analysis methods to enhance transparency and reproducibility. Additional details have been included to address potential queries.

**Results:** Some paragraphs in the results section need to be rewritten or clarified for better comprehension.

**Response:** Some paragraphs in the Results section of the revised manuscript, such as lines 469 to 513 on pages 22-24 and lines 610 to 614 on page 29 have been rewritten and clarified to improve readability and ensure accurate interpretation of the findings.

**Conclusion:** Add conclusion section with the mains finding.

**Response:** A dedicated conclusion section has been added to summarize the main findings and their implications.

We have provided detailed responses to each specific comment in the following sections, and all corresponding revisions have been highlighted in the manuscript for ease of review. We hope that the revised version meets the reviewer’s expectations. Once again, we deeply appreciate the reviewer’s thoughtful suggestions and valuable feedback.

**Comment 1:**

Line 38: Please delete Kingdom

**Response:** Thank you for pointing this out. We have deleted the term "Kingdom" as suggested. The change has been made on page 3, line 51 in the revised manuscript.

**Comment 2:**

Line 42: Parasitical plants are divided into hemiparasite and holoparasite plants according to their light and ability. Please add more details

**Response:** Thank you for your insightful comment. In response, we have expanded the section to provide more details on hemiparasitic and holoparasitic plants, including examples and their physiological characteristics. The revised text now reads as follows:

“Hemiparasites, such as *Viscum* L. (mistletoe) and *Striga* Lour. (witchweed), possess chlorophyll and are capable of limited photosynthesis, yet they still depend on their host plants for water and nutrients. In contrast, holoparasites, including *Orobanche* L. (broomrape) and *Cuscuta* L. (dodder), lack chlorophyll entirely and rely exclusively on their host plants for both carbon and mineral nutrients.”

This addition has been included on pages 3, lines 57 to 62 in the revised manuscript.

**Comment 3:**

Line 72-72: Please add citation to this sentence.

**Response:** Thank you for your suggestion. We have added the appropriate citation to support the statement as requested.

The reference is as follows:

Chen Guilin. Chinese *Cynomorium songaricum*. 1st ed. Hohhot: Science Press, 2016.

These citations have been added on page 5, lines 97 in the revised manuscript [14].

**Comment 4:**

Line 80: Please put proper word instead management.

**Response:** Thank you for pointing this out. We have replaced the word "management" with more appropriate terminology to enhance clarity and precision. We have changed “These components are known to offer benefits such as antioxidant and antiviral properties, besides helping in the management of obesity, diabetes, Alzheimer's disease, and enhancing memory function” to “These components are known to possess antioxidant and antiviral properties, as well as to aid in the prevention and treatment of obesity, diabetes, Alzheimer's disease, and enhancing memory function”.

This change has been made on page 5, lines 102-105 in the revised manuscript.

**Comment 5:**

Line 87: Delete “Our”

**Response:** Thank you for your suggestion. We have removed the term "Our" from the manuscript as requested.

This change has been made on page 6, line 112 in the revised manuscript.

**Comment 6:**

Line 114: Please add the name of three sites were research was conducted with a brief description of each one; Please add a map showing the three sites.

**Response:** Thank you for your suggestion. In response, we have added the names and descriptions of the three research sites, including their coordinates and brief ecological characteristics as follows:

“Study site 1: The research base in Jilan Tai Town, Alxa Left Banner, located in the southeastern Tengger Desert (N39°36', E105°05'), with an elevation of 1200-1400 m. This site is characterized by arid conditions and a diverse plant community, with *C. songaricum* occurring naturally and parasitizing *Nitraria* L. as its host; Study Site 2: The research base in Yinggen Sumu, Alxa Left Banner, located in the northeastern Tengger Desert (N38°27', E105°23'), with an elevation of 1200-1400 m. Similar to site 1, this region features semi-arid conditions and supports wild populations of *C. songaricum*, which parasitize *Nitraria* L.; Study Site 3: The research base in Ejina Banner, Alxa League, situated in the northwestern Badain Jaran Desert, with an elevation ranging from 1200 to 1700 m. This site is characterized by harsh desert conditions and sparse vegetation, with *C. songaricum* growing as a wild species and parasitizing *Nitraria* L.”

S1 Fig shows the locations of these three research sites. However, due to map protection, the editor has suggested that we delete the map. As a result, this part of the content cannot be presented as an image. We have provided the research site information in the form of an attached table (S1 Table).

These changes have been incorporated on pages 7-8, lines 148 to 160 in the revised manuscript.

**Comment 7:**

Line 115: What do you mean by Sutra?

**Response:** Thank you for pointing this out. To avoid any confusion, we have deleted the term "Sutra" from the manuscript.

This change has been made on page 7, line 148 in the revised manuscript.

**Comment 8:**

Line 117-120: The *C. songaricum* population is a wild population that relies on Nitrariaceae as its host plant. The *C. songaricum* seeds were in contact with or closely located in the ground, suggesting that they could be consumed by animals that live near to the ground” please remove this sentence from Materials and Methods to Introduction.

**Response:** Thank you for your suggestion. We have moved the sentences as requested, transferring them from the Materials and Methods section to the Introduction for better contextual alignment. We have inserted this sentence into page 6 lines 115 to 117 of the Introduction.

**Comment 9:**

Line 121: Please update recent citation. Why did you choose remote camera for investigation? I think investigation took a long time? Can you explain why

**Response:** Thank you for your insightful comments and questions. We have updated the citation with more recent references, as requested. These updates have been incorporated on page 9, line 184 in the revised manuscript.

References:

Monteza-Moreno CM, Rodriguez-Castro L, Castillo-Caballero PL, Toribio E,Saltonstall K. Arboreal camera trapping sheds light on seed dispersal of the world’s only epiphytic gymnosperm: *Zamia pseudoparasitica*. Ecology and Evolution. 2022; 12: e8769. <https://doi.org/10.1002/ece3.8769>

Campos CM, Velez S, Miguel MF, Papú S, Cona MI. Studying the quantity component of seed dispersal effectiveness from exclosure treatments and camera trapping. Ecology and Evolution. 2018; 8: 5470-5479. <https://doi.org/10.1002/ece3.4068>

Regarding the use of remote cameras, we chose them to monitor animal activity, particularly whether animals like mice, rabbits, and yellow sheep were gnawing on *C. songaricum*. Infrared cameras also helped observe animal visits at night. No visits were observed, which minimized field disturbance.

The extended investigation duration can be explained by:

Remote Locations: The study sites in the Tengger and Badain Jaran Deserts are difficult to access.

Weather Conditions: Extreme temperatures and seasonal rainfall limited fieldwork.

Data Collection: Long-term monitoring and sampling for phenological studies were time-consuming.

Ecological Factors: Complex interactions between *C. songaricum* and its host plant, Nitraria, required detailed, seasonal observations.

Resource Constraints: Limited resources also contributed to the longer duration.

These factors explain why the study took longer than expected.

**Comment 10:**

Line 128: Please add measures under bars for photos.

**Response:** Thank you for your suggestion. We have added the requested measures under the bars for the photos as per your recommendation. The revised figures now include the appropriate scale bars for better clarity and understanding of the data presented.

**Comment 11:**

Line 131: Throughout, use the species name of studied insects instead of beetles and ants.

**Response:** Thank you for pointing this out. We have replaced the generic terms "beetles" and "ants" with the specific species names throughout the manuscript. The revised text now refers to *Mantichorula semenowi* Reitter, 1888 (beetles) and *Messor desertora* He & Song, 2009 (ants).

**Comment 12:**

Line 133: To avoid repetition put “interactions between *C. songaricum* and the two studied insects”. Instead of “, interactions between *C. songaricum* and beetles, and *C. songaricum* and ants”

**Response:** Thank you for your suggestion. We have revised the sentence to avoid repetition, as recommended. We have modified the sentence accordingly from “interactions between *C. songaricum* and beetles, and *C. songaricum* and ants were assessed by their activity times and behaviors” to “interactions between *C. songaricum* and the two studied insects were assessed based on their activity duration and behaviors”.

This change has been made on page 10, line 220 in the revised manuscript.

**Comment 13:**

Line 135-141: Please add more details concerning beetles foraging

Experiment design, indicate the following information:

The number of visits you did in 2021 and 2022

The frequency of visits (weekly, Monthly...etc)

Did you visit the three sites during one day or each day you visit one site.

Duration of each visit to catch coleoptera. Did you use a standard duration?

Were beetles captured equally in the three sites? indicate the differences if any

**Response:** Thank you for your suggestions. Here's a more concise and direct response to your comment:

From 2021 to 2022, we conducted systematic observations at three research sites from June to August each year. Each site was observed three times per year, totaling nine observations annually, with one observation per month on average. Each site observation lasted 2-3 days, and subsequent site visits occurred only after completing observations at the previous site. The observation period for beetles and ants was consistent, spanning from sunrise to sunset for comprehensive data collection. Beetle captures were consistent across the three sites, with no significant differences observed. These details have been added to the Materials and Methods section (S2 Table).

This change has been made on pages 10-12, lines 211 to 247 in the revised manuscript.

**Comment 14:**

Line 146-147: Please mention the number of repetition to measure this duration.

How did you calculate the number of seeds carried out the anthill?

**Response:** Thank you for your comment. Here's the revised response with added clarity and conciseness:

Throughout the experiment, observations were conducted continuously from sunrise to sunset over three consecutive days at each research station, repeated three times across the study period, totaling nine days of observation. Two researchers tracked the movement of seeds by ants, noting every ant that began nibbling on seeds and eventually transporting them to the nest. The time it took for each ant to carry one seed from the *C. songaricum* flowers to the nest was recorded as a valid observation.

For calculating the number of seeds transported, random sampling was used. Each day, 10 ants were randomly selected from the ant groups visiting the *C. songaricum* plants. The carrying process of each ant was followed from the point of seed handling to entry into the nest, with the time recorded. The average time for ants to carry seeds to the nest was then calculated. By factoring in the overall frequency of ant visits and the total duration of their activity, the number of seeds transported to the nest was determined.

Specific details can be found in the Materials and Methods and Results section (S3 Table). This change has been made lines 236 to 247 on pages 11-12 and lines 442 to 445 on page 21 in the revised manuscript.

**Comment 15:**

Line 135-147: Please remove all results from this section.

**Response:** Thank you for your suggestion. We have removed all results in this section as requested. The relevant results have been supplemented in the Results section.

This change has been made on page 20, lines 437 to 445 in the revised manuscript.

**Comment 16:**

Line 152-153: Please rephrase the sentence: 30 beetles captured in the wild were placed in the same breeding room in groups of 10, for a total of three breeding rooms (n=30).

**Response:** Thank you for your suggestion. We have rephrased the sentence for clarity as follows:

Mixed rearing: Thirty *M. semenowi* captured from the wild were placed in three independent rearing rooms, with 10 *M. semenowi* in each room (The rearing room is a transparent cuboid, measuring 20 cm in length, 10 cm in width and 15 cm in height. Its top is fitted with a grid cover, facilitating gas exchange and observation).

This change has been made on page 13, lines 284 to 287 in the revised manuscript.

**Comment 17:**

Line 148-165: Please mention breeding parameters in laboratory for both ants and coleoptera.

**Response:** Thank you for your suggestion. We have added the following information regarding the breeding parameters in the laboratory:

"Both *M. semenowi* and *M. aciculatus* were subjected to feeding experiments in a laboratory setting under controlled conditions. Specifically, for *M. semenowi*, the breeding chamber was maintained at a constant temperature of 25 ± 2°C, with relative humidity ranging from 40% to 60%. For *M. aciculatus*, the artificial nests were kept under the same temperature conditions, with humidity precisely controlled between 50% and 60%. During the experiment, both species had unrestricted access to water, and *C. songaricum* seeds were the sole food source."

This additional information has been included in the Materials and Methods section for clarity. This change has been made on page 13, line 267 to 273 in the revised manuscript.

**Comment 18:**

Line 181-192: Please mention the number of repetitions adopted in experiment of odor and view signals.

**Response:** Thank you for your insightful comment. To address this, we have provided the details regarding the number of repetitions for the odor and view signal experiments in the revised manuscript.

The experiment was conducted with two treatment groups, each repeated for 7 consecutive days, as follows:

“Two treatment groups were set up in the experiment, each repeated for 7 consecutive days. The number and types of visitors were recorded daily from sunrise to sunset. Specifically, we tested the visitors' response to the fruit sequence (view signals) and seed scent (odor signals). Two treatment groups were set up in the wild. Treatment A involved covering the infructescence with yellow gauze mesh bags to isolate the visual signal (color) of *C. songaricum*, while the control group was covered only with yellow mesh bags to eliminate material interference (to test the visitors’ response to *C. songaricum* odor signals); Treatment B involved the infructescence was covered with a transparent plastic bag to block the scent of *C. songaricum* seeds, while the control group was only covered with plastic bags to eliminate material effects (to test the visitors' response to *C. songaricum* view signals).”

The study was carried out at three research sites, and the types and numbers of visitors were recorded daily from sunrise to sunset (6:00 am to 8:00 pm). The experiment was repeated for 7 consecutive days to ensure the reliability and consistency of the results.

These updates have been incorporated into the Materials and Methods section for further clarity.

This change has been made on pages 18-19, lines 385 to 401 in the revised manuscript.

**Comment 19:**

Line 192: Before results section put a paragraph of statistic’s analysis. Mention the tests used.

**Response:** Thank you for your valuable feedback. In response to your suggestion, we have added a statistical analysis section to the paper, which reads as follows:

“A direct comparison was made of the visiting time and frequency of each visitor (*M. Semenowi* and *M. aciculatus*) to *C. songaricum*. We used Origin 2022 to construct graphs and tables displaying the visiting frequency and duration to evaluate the proportional contributions of *M. Semenowi* and *M. aciculatus* among the visitors to *C. songaricum*. To explore the impact of the studied species on *C. songaricum* seeds, we conducted a statistical analysis using SPSS 26 software. Prior to further analysis, the Shapiro-Wilk test was applied to assess the normality of the data and evaluate the homogeneity of variance (ANOVA). The following data were analyzed: 1) Seeds collected by *M. Semenowi* at three time points under different rearing modes; 2) Seeds collected from *M. aciculatus* nests at three-time points. The mean and standard deviation of the number of seeds consumed by *M. Semenowi* and *M. aciculatus* at each time point were calculated. To determine whether *C. songaricum* attracts visitors via visual or olfactory signals, we used the number and frequency of visitors during different time periods in the field experiment to generate bar charts using Origin 2022.” This change has been made on page 19-20, lines 408 to 427 in the revised manuscript.

**Comment 20:**

Line 197: Throughout, use the species name of studied insects instead of beetles and ants.

**Response:** Thank you for your comment. We have replaced the generic terms "beetles" and "ants" with the specific species names throughout the manuscript, as requested.

**Comment 21:**

Line 199: To avoid repetition please rephrase the sentence “the seeds are often visited by ants, and ants usually carry the seeds to their nests.

**Response:** Thank you for your suggestion. We have modified the sentence from “the seeds are often visited by ants, and ants usually carry the seeds to their nests” to “*M. aciculatus* frequently visited the plant and carried seeds back to their nests”.

This change has been made on page 20, lines 440-441 in the revised manuscript.

**Comment 22:**

Line 204: Please rephrase the title of table1. Delete the name of studied sites in the title

**Response:** Thank you for your suggestion. We have rephrased the title of Table 1 by removing the names of the studied sites, as requested. The updated title now reads:

Table 1: Identity of *C. songaricum* seed consumers and the duration of their visits within a 24-hour period.

This change has been made on page 21, line 451 in the revised manuscript.

**Comment 23:**

Line 219: Fruit or seeds? If fruit, how many seeds in average contain?

**Response:** Thank you for highlighting the issue. We are sorry that we made a mistake in our manuscript. We are referring to the seed of *C. songaricum* and have corrected “fruit” to “seed”.

This change has been made on page 22, line 474 in the revised manuscript.

**Comment 24:**

Line 222: What do you mean by “most recently 20 cm”?

**Response:** Thank you for your comment. The phrase "most recently 20 cm" has been clarified to indicate that it refers to the straight-line distance observed in the field between the beetle's nest and its feeding site. We have re-described it as follows:

“Our observations revealed that the *M. semenowi* constructed their nests up to 50 centimeters away from the feeding site, which is the *C. songaricum* infructescence. *M. semenowi* forage near the *C. songaricum* infructescence”.

This change has been made on page 22, lines 477 to 479 in the revised manuscript.

**Comment 25:**

Line 225: How did you calculate 47.4%. You didn’t mention the protocol in Material & methods section?

**Response:** Thank you for your comment. We collected 347 fecal samples from 150 beetles captured in the wild. Of these, 178 samples contained intact seeds, which were produced by the defecation of 71 beetles. This data allowed us to calculate the percentage of fecal samples containing intact seeds, which amounted to 47.4%. For further clarification, a detailed description of this procedure has been added to the Materials and Methods section.

This change has been made lines 223 to 250 on pages 11 to 13 in the revised manuscript.

**Comment 26:**

Line 228: There is a contradiction between the number of seeds found in excrement and the maximum number of seeds eaten by beetles (line 217)

**Response:** Thank you for pointing out the contradiction. The average number of seeds found in the excrement (14.7 seeds) refers to beetles captured in the wild. Since we could not determine their prior feeding history, this figure includes seeds they had ingested up until capture. During the experiment, we closely monitored the beetles until they stopped defecating, and no additional damaged seeds were found in the feces. This observation allowed us to calculate the average number of seeds ingested. Additionally, under controlled feeding conditions, further experiments revealed that the maximum number of seeds beetles can ingest per day is 12. To address the contradiction, we have removed the earlier mention of the 14.7 average from the manuscript.

**Comment 27:**

Line 229-238: You analyzed the obtained data in global manner (you compare only rate of excreted seeds); Please rewrite this paragraph to mention statistical test used and how you performed analysis; You should compare the number of seeds excreted between the two feeding systems adopted (individual and mixed breeding) while considering beetles as repetition.

**Response:** Thank you for your valuable feedback. In response to your comment, we have rewritten the paragraph to specify the statistical methods used and to compare the number of excreted seeds between the two rearing systems (individual and mixed) while considering beetles as repetitions.

We performed a detailed analysis using a two-sample t-test to compare the number of seeds excreted in both rearing modes. Our results showed no significant difference in the total number of seeds collected: 286 ± 3 for individual rearing and 289 ± 6 for mixed rearing (n = 300). Furthermore, beetles excreted a significant number of seeds within 12 hours, with 65.04% (186 ± 3) for individual and 57.79% (167 ± 5) for mixed rearing (n = 286 and n = 289, respectively). A paired t-test confirmed no significant difference in the excretion rate between the two rearing modes.

We have revised the manuscript accordingly to clarify these statistical methods and provide a more accurate analysis.

This change has been made on Pages 23-24, Lines 490 to 513 in the revised manuscript.

**Comment 28:**

Line 239: Please make the table title short as possible.

**Response:** Thank you for your suggestion. The title of Table 2 has been shortened for conciseness and now reads:

Table 2. Intact *C. songaricum* seeds in beetle feces under different feeding modes.

This change has been made on Page 24, Line 524 in the revised manuscript.

**Comment 29:**

Line 243: Table 2: you mentioned in experiment that beetles reared individually were feed only 10 seeds. So, I think ungnawed seeds and gnawed seeds will be less than 10. Please how did you calculate the number of seeds in beetle feces under different feeding system?

**Response:** Thank you for your comment and for pointing out the confusion. It's not 10 seeds per beetle but rather 10 seeds per beetle for a total of 30 beetles, making the total number of seeds fed 300. For calculating the number of seeds in beetle feces under different feeding systems, they collect beetle feces in four stages. After collection, they use a stereomicroscope to carefully screen out *C. songaricum* seeds through inspection to conduct the relevant data statistics and analysis. The detailed process has also been supplemented and improved in the "materials and methods" section.

**Comment 30:**

Line 252: Please replace “the beetles 24 h after feeding, the seeds are feces” by the beetles 24 h after feeding, the seeds are found in feces

**Response:** Thank you for your suggestion. We have modified the sentence accordingly from “the beetles 24 h after feeding, the seeds are feces. It was found in the seeds that the external style of the seeds had been completely consumed, and all the *C. songaricum* seed coat was exposed, and the surface was smooth” to “24 hours after feeding, the seeds found in feces showed that external style had been completely consumed, with the entire seed coat exposed and the surface smooth”.

This change has been made on page 25, lines 539-542 in the revised manuscript.

**Comment 31:**

Line 257: Please delete (and exposed)

**Response:** Thank you for your suggestion. We have removed the phrase "(and exposed)" from the manuscript as requested.

This change has been made on page 26, line 546 in the revised manuscript.

**Comment 32:**

Line 258-265: Please move this paragraph to discussion section.

**Response:** Thank you for your comment. In response, we have moved the paragraph to the Discussion section, as suggested.

This change has been made on page 34, lines 726 to 734 in the revised manuscript.

**Comment 33:**

Line 271: Please mention the dissection experiment in Methods section

**Response:** Thank you for your comment. We have added the anatomical test details to the Methods section. This change has been made on pages 15-16, lines 328-343 in the revised manuscript.

**Comment 34:**

Line 310-314: Please rewrite this paragraph to avoid repetition of some words.

**Response:** Thank you for your suggestion. We have revised the paragraph to reduce repetition and improve readability. The revised paragraph now reads:

Using stereoscopic microscopy, we observed that the seed coats of *C. songaricum* seeds in the *M. aciculatus* nests remained intact throughout the 6 to 48 h period. *M. aciculatus* primarily consumed the styles attached to the seeds. At 6 and 12 h, some seeds retained intact styles, while by 24 h, none of the seeds had complete appendages.

This change has been made on page 29, lines 610 to 614 in the revised manuscript.

**Comment 35:**

Line 335-351: Please mention how did you calculate the number of visit? And for how long? I recommend the authors to calculate number or the frequency of visits for each insect, and then compare the number of visits between the two visitors. Please mention how did you performed statistical analysis and which test did you used

**Response:** Thank you for your helpful suggestion. We have updated the manuscript to clarify the calculation of visit numbers and the statistical analysis performed.

Field observations were conducted to record the number of visits by beetles and ants to *C. songaricum*. A visit was defined as any interaction between the insect and the plant. The visit frequencies in each group were as follows:

Fully exposed: 10 ± 1.99 (n=257); Odor treatment: 7.7 ± 0.52 (n=204); Odor control: 3.82 ± 0.32 (n=112); Visual treatment: 5.63 ± 0.82 (n=136); Visual control: 2.90 ± 0.18 (n=87). We used ANOVA to compare visit numbers across the groups, with post hoc tests to further explore the differences. The results showed that the fully exposed group had significantly more visits than all other groups (*p* < 0.01), and there were significant differences between treatment and control groups (*p* < 0.05). However, no significant difference was observed between the odor control and visual control groups. These findings suggest that the odor of *C. songaricum* plays a primary role in attracting visitors, while color has a secondary role.

The details have been listed in the “Materials and Methods” and “Results” section of the manuscript.

**Responses to Reviewer #2**

We sincerely thank Reviewer #2 for the thorough review and insightful comments, which have greatly improved the quality of our manuscript. Below, we provide a detailed point-by-point response to address each of the suggestions and concerns raised:

**A. Linguistic Revision**

**Response:** We acknowledge the reviewer and have thoroughly revised the manuscript to enhance its clarity and readability. Professional language editing services are used to ensure the highest linguistic quality.

**B. Scientific Name of the Beetle**

**Response:** The scientific name of the beetle has been determined as *Mantichorula semenowi* Reitter, 1888. This information has been incorporated into the manuscript for clarity and accuracy.

**C. Clarification of Ant Species Name**

**Response:** We appreciate the reviewer pointing out the discrepancy regarding *Messor desertus*. After further investigation and consultation with relevant resources, we confirm that the species is *Messor desertora* He & Song, 2009, which is valid and found in our study area. This has been corrected and clarified in the revised manuscript, and the proper citation of the species name, including the author and year, has been provided.

**D. Protocol for Ant Collection**

**Response:** Details regarding the protocol for ant collection and their transfer to artificial nests have been added to the Materials and Methods section. We have also included air and surface soil temperatures recorded during collection to provide a comprehensive context for the procedures.

**E. Temperature Data**

**Response:** We acknowledge the importance of temperature data in understanding insect activity patterns. Soil temperatures (measured at 1 cm depth) and air temperatures corresponding to the ant activity periods presented in the results have been added to the manuscript. These data are discussed in the context of foraging behavior and activity patterns.

**F. Clarification on Ant Behavior**

**Response:** We thank the reviewer for the insightful comment regarding the behavior of ants from the genus Messor. In the revised manuscript, we have explicitly clarified that the ants feed on infructescences and seeds outside the nest. We have also elaborated on the rarity of such behavior and its ecological significance. This point has been carefully reviewed and addressed.

**G. Role of Ants in Seed Management**

**Response:** The ecological role of Messor ants in seed management has been analyzed in greater depth. We have included additional discussions on:

The destination of seeds collected by ants, including their potential consumption, storage, or disposal as waste.

The interaction between lizards, Messor ants, and seeds, and its implications for seed dispersal and germination.

The possibility of seed germination in abandoned ant nests or failed colonies, as well as potential dispersal by other agents.

These discussions have been added to the Discussion section, incorporating relevant literature and ecological perspectives.

We hope that these revisions address all the concerns raised by Reviewer #2 and improve the scientific robustness and clarity of the manuscript. We are grateful for the valuable feedback, and we look forward to further suggestions.

We have provided detailed responses to each specific comment in the following sections, and all corresponding revisions have been highlighted in the manuscript for ease of review. We hope that the revised version meets the reviewer’s expectations.

**Comment 1:**

L14: (*C. songaricum*) Not necessary, however, mention the author and the year of description.

**Response:** Thank you so much for your thoughtful and constructive suggestion. In response, we have revised the manuscript to include the author and year of description for *C. songaricum*, as per your recommendation.

This change has been made on page 1, line 18 in the revised manuscript.

**Comment 2:**

L25: No information about the name "Messor desetus" was provided in the document. In the absence of the author's name and the year of description, it is unclear which species you are referring to. This name does not appear in *An Online Catalog of the Ants of the World* by Barry Bolton. You are probably referring to *Messor desertor* He & Song, 2009, which is a valid species present in your study area.

**Response:** Thank you very much for your careful review and for pointing out this error. We truly appreciate your attention to detail. After revisiting the species names, we confirm that the correct species are (*Mantichorula semenowi* Reitter, 1888) for the beetles and (*Messor desertora* He & Song, 2009) for the ants, as stated in the manuscript.

This change has been made on page 2, lines 33-34 in the revised manuscript.

**Comment 3:**

L58: "10 familiesé. You have listed only 8 families in parentheses; kindly double-check this information. Why not add the Cytinaceae and Orobanchaceae families to the list?

**Response:** Thank you so much for your careful review and for pointing out the discrepancy. After carefully revisiting the list, we have added the Cytinaceae and Orobanchaceae families, as you kindly suggested, to ensure the information is both complete and accurate.

This change has been made on page 4, lines 79-82 in the revised manuscript.

**Comment 4:**

L60: add "and"

**Response:** Thank you for your suggestion. The word "and" has been added as requested, and the sentence has been revised accordingly.

This change has been made on page 4, line 84 in the revised manuscript.

**Comment 5:**

L65: Are you referring to Suetsugu (2018b)? If not, please specify the year of publication.

**Response:** Thank you for pointing that out. We are indeed referring to the works by Suetsugu (2018a, 2018b). The manuscript has been updated to include both citations appropriately.

This change has been made on page 4, line 89 in the revised manuscript.

**Comment 6:**

L72: genus Cynomorium from, and not C. Cynomorium.

**Response:** Thank you for your comment. The manuscript has been revised to use "genus *Cynomorium*" as suggested.

This change has been made on page 5, line 95 in the revised manuscript.

**Comment 7:**

L111: in 2021 and 2022.

**Response:** Thank you for your suggestion. The manuscript has been revised to accurately reflect "in 2021 and 2022," ensuring clarity in the timeframe mentioned.

This change has been made on page 7, line 142 in the revised manuscript.

**Comment 8:**

L113: Cite the reference for these data.

**Response:** Thank you for your comment. We have added the appropriate reference to support these data, as suggested. This change has been made on page 7, line 147 in the revised manuscript.

Reference:

Announcing the website of Alxa League. The Administrative Office of Alxa League. Alxa League: Climatic characteristics. 2025. <http://www.als.gov.cn/col/col3386/index.html>

**Comment 9:**

L 113-114: It is recommended to use the Köppen-Geiger classification (Kottek et al., 2006).

**Response:** Thank you for the helpful suggestion. We have revised the manuscript to incorporate the Köppen-Geiger classification system, as recommended, and cited the reference accordingly.

This change has been made on page 7, lines 144-145 in the revised manuscript.

**Comment 10:**

L141: (were counted (n=178, 51.29%).) This is a result and should be moved to the results section.

**Response:** Thank you for your observation. We agree that this information belongs in the Results section. The sentence '(were counted (n=178, 51.29%))' has been relocated to the Results section in the revised manuscript to ensure clarity and logical flow.

This change has been made on page 20, lines 437 to 439 in the revised manuscript.

**Comment 11:**

L150: Can you justify this duration? 48h

**Response:** Thank you for raising this important question. The 48-hour fasting period was chosen based on established methodologies in insect behavioral and physiological studies. According to Lundgren JG & Gilad T, a similar fasting duration allows sufficient time for the beetles to fully digest any previously consumed food, ensuring they are in a post-fasting state. This practice minimizes potential biases from prior foraging activity, providing a reliable basis for assessing their responses to *C. songaricum* seeds during the feeding trials. Additionally, in our experiment, the beetles were fasted for 24 h and were unable to empty their digestive tract, further supporting the appropriateness of this duration.

The reference has been cited in the revised manuscript on page 13, line 279.

References:

Lundgren JG, Seagraves MP. Physiological benefits of nectar feeding by a predatory beetle. Biological Journal of the Linnean Society. 2011; 104: 661-669. <https://doi.org/10.1111/j.1095-8312.2011.01729.x>

Gilad T, Koren R, Moalem Y, Subach A, Scharf I. Effect of continuous and alternating episodes of starvation on behavior and reproduction in the red flour beetle. Journal of Zoology. 2018; 305: 213-222. https://doi.org/10.1111/jzo.12556

**Comment 12:**

L160-161: This section requires greater detail. How many foragers did you collect? How did you ensure they belonged to the same colony (to avoid conflicts)? Were they collected directly from the plant or during their return to the nest? Was there a foraging column present? How was the artificial nest designed? Are there any photos available (as supplementary material)?

**Response:** Thank you for your detailed questions. In this study, we collected 150 foraging ants from the wild to ensure they all belonged to the same colony and avoided conflicts. We used a vacuum device to capture ants foraging on plants within a 1.5 to 2-meter radius from the collection site, ensuring they came from the same nest group. To avoid disturbing their natural behavior, we refrained from collecting ants during their return to the nest. Our laboratory observations showed that the collected ants did not display seed transport behavior. We speculate that the absence of queen ants or key environmental cues may have inhibited this behavior, based on relevant literature.

To ensure experimental consistency, we collaborated with a biotechnology company to obtain ant populations from the same region. Three sets of ants were provided, each consisting of one queen ant, 120 worker ants, and 10 soldier ants, which were used to establish artificial nests for further behavioral studies.

For the artificial nest design, we purchased specialized nests online, specifically made for ant experiments. These nests closely resemble the ants' natural habitat, ensuring the accuracy and reproducibility of the experiments. The revised details have been incorporated into the manuscript on pages 14-15, lines 304 to 325.

**Comment 13:**

L161-163: We fed them 100 *C. songaricum* seeds. After 6 h, 12 h, 24 h, and 48 h of observations, we collected the *C. songaricum* seeds from the ant nests and counted the number of intact seeds (n=300).

**Response:** Thank you for your observation. We have revised the corresponding section to clarify the procedure and improve the accuracy of the description, ensuring it accurately reflects the experimental timeline and the collection of *C. songaricum* seeds.

This change has been made on page 14, lines 295 to 297 in the revised manuscript.

**Comment 14:**

L167: due to chewing processes

**Response:** Thank you for pointing this out. We have revised the phrasing for clarity to better reflect the chewing processes. We have changed the sentence from “Mechanical damage to seeds due to chewing or chewing processes is a common phenomenon in many plant species” to “Mechanical damage to seeds caused by chewing is common phenomenon in many plant species”.

This change has been made on page 17, line 363 in the revised manuscript.

**Comment 15:**

L170: We then used

**Response:** Thank you for pointing this out. We have revised the phrasing for clarity to ensure a more precise description.

This change has been made on page 17, line 372 in the revised manuscript.

**Comment 16:**

L173: De Vega and De Oliveira

**Response:** Thank you for highlighting this. The formatting of 'De Vega and De Oliveira' has been corrected as suggested.

This change has been made on page 18, line 376 in the revised manuscript.

**Comment 17:**

L179: greasy smell?

**Response:** Thank you for your observation. The term has been revised to 'greasy odor' in the manuscript for consistency.

This change has been made on page 18, line 383 in the revised manuscript.

**Comment 18:**

L182: The infructescence was covered

Response: Thank you for pointing this out. We have revised the phrasing for clarity to improve the description. We have modified the sentence accordingly from “**Treatment A:** The infructescence were covered with yellow gauze mesh bags to eliminate interference from the color of *C. songaricum* itself. The control group only had yellow gauze mesh bags to eliminate the influence from experimental materials” to “**Treatment A** involved covering the infructescence with yellow gauze mesh bags to isolate the visual signal (color) of *C. songaricum*, while the control group was covered only with yellow mesh bags to eliminate material interference”.

This change has been made on page 18, lines 390-394 in the revised manuscript.

**Comment 19:**

L185: was cavered

**Response:** Thank you for pointing this out. We have revised the phrasing for clarity to improve the description. We have changed “is covered” to “was covered”.

This change has been made on page 18, line 396 in the revised manuscript.

**Comment 20:**

L192: from 6:00 am to 8:00 pm

**Response:** Thank you for pointing this out. The phrasing has been revised for clarity to specify the time range from 6:00 am to 8:00 pm.

This change has been made on page 19, line 406 in the revised manuscript.

**Comment 21:**

L196: They visit the plant, not the seeds!

**Response:** Thank you for pointing this out. We appreciate your keen observation, and the text has been revised to clearly indicate that the ants visit the plant, not the seeds.

This change has been made on page 20, line 433 in the revised manuscript.

**Comment 22:**

L198: Under what weather conditions? Specifically, at what temperature? Temperature is the primary factor determining the foraging activity of ants, not the availability of seeds.

**Response:** Thank you for your insightful comment. The duration of ant and beetle feeding within a *C. songaricum* cluster is 10-12 hours under weather conditions with temperatures ranging from 16 to 36°C. Detailed information on the specific timing and temperature of ant foraging activities is provided on Results in the manuscript. This change has been made on page 20, line 437 in the revised manuscript.

**Comment 23:**

L201-203: Here too, it is essential to have data on nocturnal temperatures for the same reason mentioned above.

**Response:** Thank you for your valuable feedback. We have now included data on nocturnal temperatures (≥ 10 ± 3°C) in page 21, line 450.

**Comment 24:**

L205: situation ????

**Response:** Thank you for pointing this out. Based on the comments raised by both reviewers, we have deleted this paragraph.

**Comment 25:**

L206: study

Table1: Coleptera, not in italics. Myrmicinae, not in italics, desertus in italics (after cheking the name). No information about the name "Messor desetus" was provided in the document. In the absence of the author's name and the year of description, it is unclear which species you are referring to. This name does not appear in *An Online Catalog of the Ants of the World* by Barry Bolton. You are probably referring to *Messor desertor* He & Song, 2009, which is a valid species present in your study area.

**Response:** Thank you for your careful review. Due to a number of issues with this paragraph, we have removed this description.

**Comment 26:**

L210: Close the parenthesis.

**Response:** Thank you for your comment. The parenthesis has been closed as requested in the revised manuscript.

This change has been made on page 21, line 458 in the revised manuscript.

**Comment 27:**

L211: deleta Celsius.

**Response:** Thank you for your suggestion. The term "Celsius" has been deleted as requested in the revised manuscript.

This change has been made on page 22, line 459 in the revised manuscript.

**Comment 28:**

L211: beetle activity decreases

**Response:** Thank you for pointing this out. We have revised the phrasing for clarity. We have changed “Beetle activity decreases” to “*M. semenowi* activity decreases”.

This change has been made on page 22, line 460 in the revised manuscript.

**Comment 29:**

L216: feeds on

**Response:** Thank you for pointing this out. The phrasing has been revised to “feeds on” for clarity. We have modified “beetle eat” to “*M. semenowi* feeds on”.

This change has been made on page 22, line 469 in the revised manuscript.

**Comment 30:**

L229: of C. songaricum

**Response:** Thank you for pointing this out. We have reworded the paragraph and removed the offending sentence.

**Comment 31:**

L230: the analysis

**Response:** Thank you for pointing this out. We have reworded the paragraph and removed the offending sentence.

**Comment 32:**

L236: 48 h after the beetles had eaten

**Response:** Thank you for pointing this out. We have reworded the paragraph and removed the offending sentence.

This change has been made on page 24, lines 506-508 in the revised manuscript.

**Comment 33:**

L237-238: There was no significant difference between the two feeding modes.

**Response:** Thank you for pointing this out. We have revised the phrasing for clarity. We have modified the sentence accordingly from “The difference was not significant under different feeding modes” to “there was no significant difference in the total number of intact *C. songaricum* seeds excreted by *M. semenowi* under the two rearing modes”.

This change has been made on page 24, lines 508-510 in the revised manuscript.

**Comment 34:**

L288: (a-c) Anatomy

**Response:** Thank you for your comment. The format has been modified as suggested.

This change has been made on page 27, line 582 in the revised manuscript.

**Comment 35:**

L290: (j-l) Anatomy

**Response:** Thank you for your comment. The format has been modified as suggested.

This change has been made on page 28, line 584 in the revised manuscript.

**Comment 36:**

L294-295: This requires specifying the temperature ranges that correspond to this activity.

**Response:** Thank you for your valuable feedback. We have now specified the temperature range corresponding to this activity in the revised manuscript (16℃~36℃, ±3℃).

This change has been made on page 28, line 588 in the revised manuscript.

**Comment 37:**

L198-299: When reading this text, it appears that the ants feed on the infructescences of seeds or the infructescences of *C. songaricum* outside the nest, especially when you mention that the midday heat forces them to return to their nests. If this is the case, it would be better to state it explicitly, as it is well known for the genus *Messor* that the consumption of seeds outside the nest is very rare. Their behavior is primarily focused on transporting and storing seeds for internal processing and consumption. It is therefore crucial to verify this information.

**Response:** Thank you for your thoughtful comment. We have carefully reconsidered the description of the ants' behavior. The ants indeed feed on the infructescences of *C. songaricum* outside the nest, and we agree with your observation that consumption of seeds outside the nest is rare for *Messor aciculatus* Nicolai, 1840. Their primary behavior is focused on transporting and storing seeds. We have revised the manuscript to explicitly state this and ensure the accuracy of the information. We appreciate your attention to this important detail.

**Comment 38:**

L306-310: This should be mentioned in the Materials and Methods section.

**Response:** Thank you for your suggestion. We have now included this information in the Materials and Methods section as recommended. This change has been made on page 15, lines 310 to 325 in the revised manuscript.

**Comment 39:**

L312: and ants consumed only the style attached to the seeds.

**Response:** Thank you for your comment. The phrasing has been revised to clarify that the ants consumed only the style attached to the seeds. We have changed the sentence from “and the ants only ate the style outside the *C. songaricum* seeds” to “*M. aciculatus* primarily consumed the styles attached to the seeds”. This change has been made on page 29, line 612 in the revised manuscript.

**Comment 40:**

Table 3: Seed type

**Response:** Thank you for pointing this out. We have revised the phrasing for clarity.

This change has been made on page 30.

**Comment 41:**

L373: The role of ants from the genus Messor in seed management requires an in-depth analysis of their ecological impacts. Unlike beetles, it is essential to specify the destination of the seeds collected by these ants. Lizards, acting as tertiary dispersal agents (Fig. 6), can consume *Messor* ants, raising the question of the impact of this interaction on seed germination, particularly if the seeds are ingested by the ants themselves—something that remains uncertain. Additionally, the seeds collected might be consumed by the ants (by grinding them), especially during periods of food scarcity, or discarded with waste outside the nest, where they could be dispersed by other means or germinate in situ. Finally, it would be relevant to investigate their ability to germinate in abandoned ant nests or in failed colonies.

Moreover, the microclimatic conditions in the storage chambers of the nests, such as temperature, humidity, and exposure to microbial agents, may also influence seed viability. These interactions could promote germination by altering the seed coat or, conversely, accelerate seed deterioration. These complex processes, involving seed manipulation, environmental conditions, and ant behavior, deserve further study to better understand the ecological role of Messor ants as seed dispersers and managers.

**Response:** Thank you for your thorough and insightful comment. We agree that the role of ants from the genus *M. aciculatus* in seed management is complex and requires further exploration. In the revised manuscript, we have clarified the destination of the seeds collected by the ants and discussed the potential ecological impacts, including interactions with lizards and other factors such as seed consumption, waste disposal, and the impact of the microclimatic conditions in the nests. We have also highlighted the need for future studies to investigate the germination potential of seeds in abandoned ant nests or failed colonies. We appreciate your valuable suggestions, which have greatly improved the depth of our analysis on this topic.

**Comment 42:**

L399: "to eat", As already mentioned: collecting seeds is one thing, but feeding on them on-site is another.

**Response:** Thank you for your valuable comment, and I apologize for any confusion caused by the wording. We have clarified in the revised manuscript that the ants did not feed on the seeds of *C. songaricum*. Instead, they only consumed the attached structures on the outside of the seeds, specifically the 'oil bodies'. The ants carried the seeds and, at most, nibbled on the 'oil bodies' but did not feed on the seeds themselves. We have modified the description of this sentence as follows：

“We found that *M. desertora* can only carry seeds that have fallen after *M. semenowi* has gnawed on the fleshy stems of *C. songaricum*. *M. desertora* takes these seeds to the nests and then consume the structures attached to the outside of the seeds” (page 35, lines 731-734).

**Comment 43:**

L401: Idem

**Response:** We sincerely appreciate the comment. I apologize for any confusion caused by the wording. We have clarified in the revised manuscript that ants do not feed on the seeds of *C. songaricum*.

This change has been made on page 35, lines 731-734 in the revised manuscript.

**Comment 44:**

L444-445: Here, you are referring to transportation, not on-site feeding. Please refer to the previous comments for clarification.

**Response:** Thank you for your comment. We have revised the manuscript to clearly specify that we are referring to transportation of seeds, not on-site feeding, as noted in previous comments. This distinction has now been made clear in the text (page 38, lines 796-798).

**Comment 45:**

L544: De Vega

**Response:** Thank you for pointing this out. We have revised the format for accuracy, as requested.

This change has been made on page 45, line 965 in the revised manuscript.

**Comment 46:**

L577: De Vega

**Response:** Thank you for pointing this out. We have revised the format for accuracy, as requested.

This change has been made on page 48, line 1026 in the revised manuscript.

**Comment 47:**

L587: De Castro-Arrazola

**Response:** Thank you for pointing this out. We have revised the format for accuracy, as requested.

This change has been made on page 48, line 1039 in the revised manuscript.
